# Awareness of Keratoconus and Its Relationship With Eye Rubbing Among the Population of the Eastern Province of Saudi Arabia

**DOI:** 10.7759/cureus.51627

**Published:** 2024-01-03

**Authors:** Abdulaziz AlSomali, Danah Almithn, Aisha Alamer, Abdullah Al-Omair, Fatimah Almuhaysin, Norah Almulhim

**Affiliations:** 1 Ophthalmology, College of Medicine, King Faisal University, Al-Ahsa, SAU

**Keywords:** health education & awareness, cornea abnormalities, cone-shaped cornea, eye rubbing, keratoconus (kc)

## Abstract

Background: Keratoconus is an eye condition where the cornea, the clear, dome-shaped front part of the eye, becomes thinner and gradually bulges outward into a cone shape. Keratoconus usually affects both eyes. The exact cause of keratoconus is unknown, but it is believed to involve a combination of genetic and environmental factors.

Aim: This study aims to assess the awareness level of keratoconus and its relation to eye rubbing among the population in the Eastern Province of Saudi Arabia.

Methods: A cross-sectional study was conducted in the Eastern Province of Saudi Arabia, involving all eligible participants via an online pre-designed questionnaire from March to June 2023. The data were collected through social media.

Results: A total of 388 eligible participants completed the study questionnaire. Participants’ ages ranged from 19 to 60 years, with a mean age of 26.2 ± 13.9 years. There were 265 (68.3%) female participants. Regarding public awareness about keratoconus among participants in the Eastern Province of Saudi Arabia, our research revealed a total of 101 (26%) participants had an overall good awareness of keratoconus, while 287 (74%) had a poor awareness level. The most reported source of information included scientific lectures (21.6%). Precisely, 316 (81.4%) of the research subjects rub their eyes primarily because of allergies (7%), strain headaches (25%), and itching (66.5%).

Conclusion: Most of the study participants have poor awareness about keratoconus and its relation to eye rubbing. Health education programs for the population should be conducted to enhance public awareness about keratoconus.

## Introduction

Keratoconus is an eye disease that affects the cornea and is a major cause of corneal transplants. It is a gradual and progressive condition that alters the shape of the cornea, leading to thinning, irregular astigmatism, and low vision [1, 2]. As the condition progresses, the central or paracentral portion of the cornea protrudes, giving it a cone-shaped appearance [3, 4]. Typically, individuals experience the onset of the disease during adolescence, and it continues until their third or fourth decade of life [4].

The development of keratoconus can be influenced by various factors, including hereditary and environmental influences [1]. First-degree family members and twins of keratoconus patients have a higher risk of developing the condition compared to the general population [5]. While several genetic variations have been linked to keratoconus, few of these associations have been consistently replicated [6]. The pathophysiology of keratoconus has been associated with environmental variables such as eye rubbing, atopy, allergic eye illness, dermatitis, and exposure to UV radiation [7-13]. Ethnicity is also considered a factor, with Asians experiencing a more severe and early onset of keratoconus than Caucasians [4].

Keratoconus is estimated to affect 1.38 people out of every 1,000 [9]. Saudi Arabia has the highest prevalence of keratoconus globally, with 4,790 cases per 100,000 individuals in the Middle East [10]. In Saudi Arabia, a study in the Najran region found 87.3 incidences of keratoconus for every 100,000 individuals, with 8.59% of the population in Taif having the condition [3, 4]. A six-year study conducted in Saudi Arabia identified keratoconus as the most common reason for corneal transplants in the Eastern Province [11].

## Materials and methods

Study design and participants

This community-based cross-sectional study targeted participants in the Eastern Province of Saudi Arabia aged 18 years and older. The recruitment involved the administration of an online questionnaire in Arabic, distributed through social media platforms from March 2023 to June 2023.

Questionnaire development and distribution

The questionnaire used in this study was previously validated in the Arabic language by Kordi et al. [14]. The survey questionnaire comprised three sections. The first section collected demographic data, including age, sex, and education level. The second section consisted of yes-or-no questions to assess eye allergies, other allergies, eye diseases, and vision problems. The third section included nine items evaluating participants' awareness of keratoconus, its causes, treatment, and reasons for eye rubbing.

The sample size of 385 participants was calculated using the formula n = z2pq/d2 for a 95% confidence level and 5% margin of error. To ensure broad participation, we employed a targeted approach through various social media platforms, strategically disseminating the survey link on channels such as Meta (previously known as Facebook), X (previously known as Twitter), and WhatsApp groups. This method allowed us to engage participants across different demographics and geographic locations within the Eastern Province of Saudi Arabia.

Informed consent and ethical approval

Before participation, participants were informed of the study's objectives, and informed consent was obtained. Confidentiality and voluntary participation were ensured. Ethical approval was obtained from the Deanship of Scientific Research at King Faisal University (approval number: KFU-REC-2023-FEB-ETHICS585) on February 15, 2023.

Data analysis

Upon data extraction, IBM SPSS version 22 (IBM Corp., Armonk, NY) was employed for data editing, coding, and importation. Two-tailed tests were used for statistical analysis, considering p-values below 0.05 as statistically significant. The total score for keratoconus awareness and knowledge was calculated by summing scores from each item. Individuals were classified as having poor awareness if their total score was less than 60% and good awareness if it was 60% or higher. Descriptive analyses were conducted for variables, and a graph was created to display information sources and knowledge levels. Pearson's chi-square test and exact probability test were used for small frequency distributions to measure participants' awareness, with results presented in cross-tabulation graphs.

## Results

Participant characteristics

The study included 388 qualified participants, with a mean age of 26.2 ±13.9 years, ranging from 19 to 60. Of the participants, 253 (65.2%) were single, and 265 (68.3%) were female. Educational qualifications varied, with 291 (75%) having university or postgraduate education, 95 (24.5%) having high school education, and others having lower education levels. Monthly income distribution revealed 191 (49.2%) participants earning less than 3,000 Saudi Riyals (SR), 96 (24.7%) earning 3,000-10,000 SR, and 101 (26%) earning more than 10,000 SR.

Regarding the participants' medical and family history, allergies were reported by 115 participants (29.6%), with skin allergies (43.5%) being the most common. A total of 213 (54.9%) participants reported having one or more eye diseases, including keratoconus (4.2%). Twenty-four participants (6.2%) had a family history of keratoconus (Table 1).

**Table 1:** Medical and family history of keratoconus and eye diseases among participants in the Eastern Province of Saudi Arabia (n=388). The percentages are calculated based on the total number of responses for each awareness item. "N" represents the total number of participants who provided responses for each specific item.

Medical Data	N	%
Do You Have Any Allergies?		
Yes	115	29.60%
No	273	70.40%
If Yes, Type of Allergy (n = 115)		
Skin Allergy	50	43.50%
Eye Allergy	31	27.00%
Chest Allergy	16	13.90%
Digestive	9	7.80%
Allergic Rhinitis	4	3.50%
Allergic Rhinitis	2	1.70%
Sinusitis	2	1.70%
Food Allergy	1	0.90%
Do You Suffer from Visual Problems or Eye Diseases?		
Yes	213	54.90%
No	175	45.10%
If Yes, What Do You Suffer From? (n = 213)		
Refractive Error	152	71.30%
Refractive Surgery Done in the Past	29	13.60%
Keratoconus	9	4.20%
Eye Surgery	8	3.80%
Using Contact Lenses for Vision Correction	6	2.80%
Amblyopia	3	1.40%
Dry Eye	2	0.90%
Retinitis Pigmentosa	2	0.90%
Cataract	1	0.50%
Glaucoma	1	0.50%
Do You Have a Family History of Keratoconus?		
Yes	24	6.20%
No	364	93.80%

Public awareness about keratoconus

Table 2 illustrates public awareness about keratoconus.

**Table 2:** Awareness of keratoconus among participants in the Eastern Province of Saudi Arabia (n=388) The percentages are calculated based on the total number of responses for each awareness item. "N" represents the total number of participants who provided responses for each specific item.

Awareness Items	N	%
Have You Ever Heard About Keratoconus?		
Yes	171	44.10%
No	217	55.90%
What Is Keratoconus?		
Corneal Thinning and Weakness	105	27.10%
Increased Corneal Thickness	82	21.10%
Corneal Inflammation	46	11.90%
Immune Disease	13	3.40%
I Don't Know	142	36.60%
There Is a Relationship Between Keratoconus and Allergy.		
Yes	178	45.90%
No	31	8.00%
I Don't Know	179	46.10%
Keratoconus Leads to Poor Eyesight.		
Yes	229	59.00%
No	5	1.30%
I Don't Know	154	39.70%
Treatment Methods for Keratoconus.		
Surgery	165	42.50%
Eyeglasses	71	18.30%
Contact Lenses	67	17.30%
Using Eye Drops	49	12.60%
No Treatment	20	5.20%
I Don't Know	184	47.40%
The Habit of Eye Rubbing Is Described As		
It May Lead to Keratoconus	164	42.30%
One of the Safe Habits	40	10.30%
I Don't Know	184	47.40%

Of the participants, 171 (44.1%) had heard about keratoconus. Specific knowledge about corneal thinning and weakness was noted by 27.1% of participants. Additionally, 178 (45.9%) were aware of the relationship between keratoconus and allergy, and 229 (59%) knew that keratoconus could lead to poor eyesight. Treatment methods were recognized by 165 (42.5%) participants, with 47.4% expressing uncertainty. A total of 164 (42.3%) participants acknowledged that eye rubbing could lead to keratoconus.

Overall public awareness and information sources

**Figure 1:**
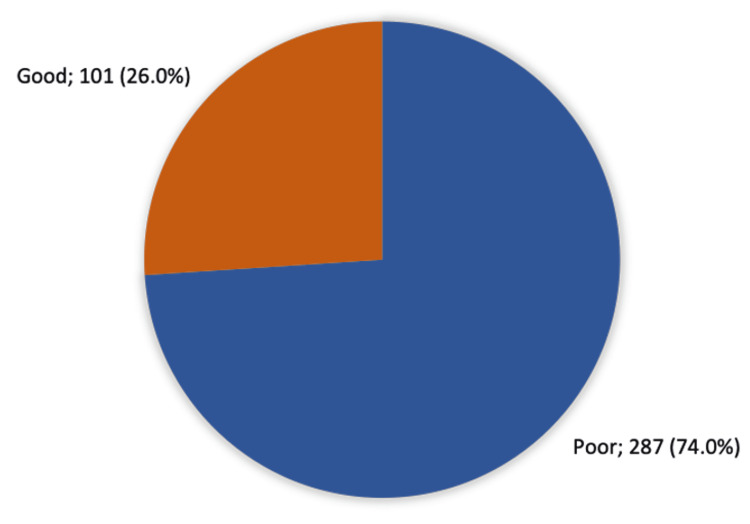
Participants' general public awareness of keratoconus in the Eastern Province of Saudi Arabia

Figure 1 reveals that 101 (26%) participants had a high level of awareness about keratoconus, while 287 (74%) had a low level of awareness.

**Figure 2:**
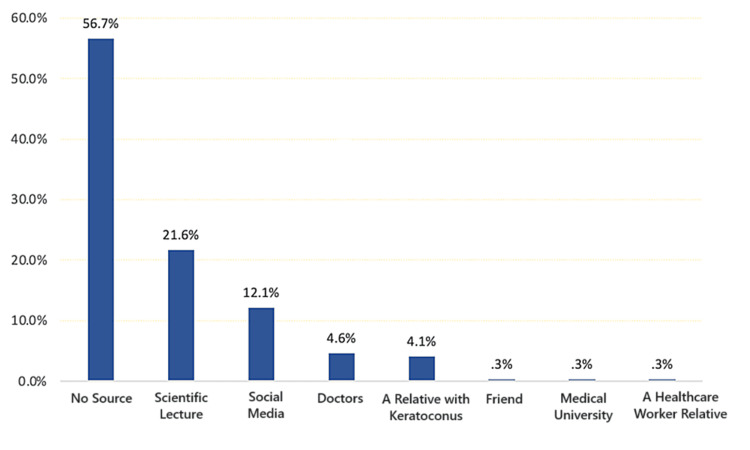
Sources of information about keratoconus among study participants in the Eastern Province of Saudi Arabia

Figure 2 displays the primary sources of information, with scientific lectures (21.6%) and social media (12.1%) being the most commonly reported sources; a significant proportion (56.7%) reported having no source of information.

Eye rubbing and sun exposure habits

Table 3 details the reasons why the participants rub their eyes and showcases their sun exposure habits.

**Table 3:** Eye care practices among the participants in the Eastern Province of Saudi Arabia (n=388) The percentages are calculated based on the total number of responses for each awareness item. "N" represents the total number of participants who provided responses for each specific item.

Practice	N	%
Do You Rub Your Eyes?		
Yes	316	81.40%
No	72	18.60%
If Yes, Why? (n=316)		
Itching	210	66.50%
Strain or Headache	79	25.00%
Allergy	22	7.00%
Eye Dryness	3	0.90%
Foreign Body	1	0.30%
Sleepiness	1	0.30%
Do You Get a Lot of Sun Exposure Throughout the Day?		
Yes	181	46.60%
No	207	53.40%
If the Answer Is Yes, Do You Wear Sunglasses?		
Yes	90	50.30%
No	89	49.70%

Of the participants, 316 (81.4%) reported eye rubbing for various reasons, including itching (66.5%) and strain headache (25%). Moreover, 181 (46.6%) were exposed to sunlight, and 90 (50.3%) used sunglasses.

Factors associated with keratoconus awareness

Table 4 explores factors associated with participants' keratoconus awareness.

**Table 4:** Factors associated with participants’ awareness of keratoconus in the Eastern Province of Saudi Arabia (n=388) The table presents factors associated with participants' awareness of keratoconus in the Eastern Province of Saudi Arabia. The columns display the number (N) and percentage (%) of participants with poor and good overall awareness levels for each factor. The statistical significance is indicated by the p-value, with a threshold of 0.05 considered significant.

Factors		Overall Awareness Level				P-Value
		Poor		Good		
		N	%	N	%	
Age (in years)	19-30	196	68.5%	90	31.5%	<0.01
	31-40	36	90.0%	4	10.0%	
	41-50	28	84.8%	5	15.2%	
	51-60	27	93.1%	2	6.9%	
Gender	Male	96	78.0%	27	22.0%	0.21
	Female	191	72.1%	74	27.9%	
Marital Status	Single	171	67.6%	82	32.4%	<0.01
	Married	116	85.9%	19	14.1%	
Scientific Qualification	Middle School	2	100.0%	0	0.0%	0.68
	High School	71	74.7%	24	25.3%	
	University/Post-graduate Education	214	73.5%	77	26.5%	
Do You Have Any Allergies?	Yes	82	71.3%	33	28.7%	0.44
	No	205	75.1%	68	24.9%	
Do You Suffer From Visual Problems or Eye Diseases?	Yes	148	69.5%	65	30.5%	0.03
	No	139	79.4%	36	20.6%	
Do You Have a Family History of Keratoconus?	Yes	13	54.2%	11	45.8%	0.02
	No	274	75.3%	90	24.7%	
Source of Information About Keratoconus	Doctors	17	94.4%	1	5.6%	0.01
	Friend	1	100.0%	0	0.0%	
	Medical University	0	0.0%	1	100.0%	
	No Source	173	78.6%	47	21.4%	
	A Healthworker Relative	0	0.0%	1	100.0%	
	A Relative With Keratoconus	11	68.8%	5	31.3%	
	Scientific Lectures or Reading	52	61.9%	32	38.1%	
	Social Media	33	70.2%	14	29.8%	

Statistically significant associations were observed, with young age (31.5% vs. 6.9% in the older age group) and single status (32.4% vs. 14.1% in married participants) correlating with higher overall awareness. Participants with visual impairments (30.5% vs. 20.6%) and those with a family history of keratoconus (45.8% vs. 24.7%) demonstrated significantly higher awareness levels. Additionally, participants informed by medical universities and healthcare workers showed good understanding, contrasting with those receiving information from friends or having no information (p =.007).

## Discussion

Keratoconus awareness is essential to increasing understanding and knowledge about keratoconus, a condition causing the cornea to gradually thin and bulge outward. Various organizations and initiatives dedicate efforts to raising awareness about keratoconus [1, 2]. The National Keratoconus Foundation (NKCF) is the world's oldest and largest organization focused on increasing awareness of keratoconus [3]. They appointed the National Association for Stock Car Auto Racing (NASCAR) driver Joey Gase as their ambassador, providing information through their "Clearly KC" campaign. Promoting awareness about keratoconus is crucial for encouraging early detection, timely treatment, and supporting affected individuals.

The purpose of this study was to evaluate awareness about keratoconus and its relationship with eye rubbing among the population in the Eastern Province of Saudi Arabia. The study revealed that over half of the participants had eye problems, mainly refractive errors, and keratoconus was reported in less than one-fifth of the participants. A lower prevalence of keratoconus was reported by Althomali et al. [4], finding that 8.5% of study participants in Taif had keratoconus, with bilateral cases among 6.6%. Similarly, numerous studies conducted globally revealed significant disparities in prevalence rates [4, 9]. Various factors contribute to these variations, including geographical location, ethnicity, participant characteristics, and diagnostic methods [10-12].

Regarding participants' awareness of keratoconus, the current study indicated that approximately 25% exhibited satisfactory overall awareness. Over half of them knew that keratoconus leads to poor eyesight. Concerning treatment, participants were most aware of surgery, followed by eyeglasses and contact lenses, with about half unaware of treatment methods. Scientific lectures, social media, and doctors were the most reported sources of information. The study found higher awareness levels among younger participants, those experiencing visual impairments or eye-related ailments, participants with a family history of keratoconus, and those informed about keratoconus through a medical university and health workers. A similar awareness level was reported in the Aseer region by Al-Amri et al. [15], documenting that 18.7% of respondents had an overall good awareness of keratoconus. Another study in Abha revealed that the majority of participants (57.5%) reported no prior knowledge or awareness of keratoconus; only 32 (8.1%) became aware through information from healthcare providers [13]. Similarly, Al Rashed concluded that there was poor public awareness about keratoconus in Saudi Arabia [16].

Concerning eye rubbing and its relation to keratoconus, the study revealed that 164 (42.3%) participants believed eye rubbing may lead to keratoconus. Alamri et al. [17] found that 33.1% of participants considered eye rubbing a harmful habit. A much lower awareness level about eye rubbing was reported by Kordi et al. [14], with only 28.9% believing it could potentially lead to keratoconus. Furthermore, a significant majority (80.4%) reported engaging in eye-rubbing behavior, primarily due to itching.

While utilizing an online questionnaire helped reach a wide population from diverse regions, a limitation of this study was the absence of assigned data collectors for each specific region in the Eastern Province. This may result in an unequal distribution of participants throughout the province, impacting the generalizability of the findings.

## Conclusions

The current study uncovered that participants had poor awareness of keratoconus, with only about one in four demonstrating knowledge about the disease. Younger individuals with a personal or family history of the disorder exhibited higher awareness levels. The lowest awareness level was observed for the nature of the disease, while better awareness was reported for associated factors and the disorder's effects on eyesight. The authors recommend enhancing public health awareness through periodic health education programs as a crucial initiative. Consequently, further research is deemed necessary to draw more reliable conclusions, utilizing larger sample sizes and encompassing diverse populations from various regions.
